# Evaluation of the side effects of poly(epsilon-caprolactone) nanocapsules containing atrazine toward maize plants

**DOI:** 10.3389/fchem.2015.00061

**Published:** 2015-10-21

**Authors:** Halley C. Oliveira, Renata Stolf-Moreira, Cláudia B. R. Martinez, Gustavo F. M. Sousa, Renato Grillo, Marcelo B. de Jesus, Leonardo F. Fraceto

**Affiliations:** ^1^Department of Animal and Plant Biology, University of LondrinaLondrina, Brazil; ^2^Department of Physiological Sciences, University of LondrinaLondrina, Brazil; ^3^International Iberian Nanotechnology LaboratoryBraga, Portugal; ^4^Department of Environmental Engineering, Universidade Estadual PaulistaSorocaba, Brazil; ^5^Department of Biochemistry, Institute of Biology, University of CampinasCampinas, Brazil

**Keywords:** atrazine, polymeric nanoparticles, nanopesticides, nanotechnology, *Zea mays*. L

## Abstract

Poly(epsilon-caprolactone) (PCL) nanocapsules have been used as a carrier system for the herbicide atrazine, which is commonly applied to maize. We demonstrated previously that these atrazine containing polymeric nanocapsules were 10-fold more effective in the control of mustard plants (a target species), as compared to a commercial atrazine formulation. Since atrazine can have adverse effects on non-target crops, here we analyzed the effect of encapsulated atrazine on growth, physiological and oxidative stress parameters of soil-grown maize plants (*Zea mays* L.). One day after the post-emergence treatment with PCL nanocapsules containing atrazine (1 mg mL^−1^), maize plants presented 15 and 21% decreases in maximum quantum yield of photosystem II (PSII) and in net CO_2_ assimilation rate, respectively, as compared to water-sprayed plants. The same treatment led to a 1.8-fold increase in leaf lipid peroxidation in comparison with control plants. However, all of these parameters were unaffected 4 and 8 days after the application of encapsulated atrazine. These results suggested that the negative effects of atrazine were transient, probably due to the ability of maize plants to detoxify the herbicide. When encapsulated atrazine was applied at a 10-fold lower concentration (0.1 mg mL^−1^), a dosage that is still effective for weed control, no effects were detected even shortly after application. Regardless of the herbicide concentration, neither pre- nor post-emergence treatment with the PCL nanocapsules carrying atrazine resulted in the development of any macroscopic symptoms in maize leaves, and there were no impacts on shoot growth. Additionally, no effects were observed when plants were sprayed with PCL nanocapsules without atrazine. Overall, these results suggested that the use of PCL nanocapsules containing atrazine did not lead to persistent side effects in maize plants, and that the technique could offer a safe tool for weed control without affecting crop growth.

## Introduction

Atrazine (6-chloro-N2-ethyl-N4-isopropyl-1,3,5-triazine-2,4-diamine) is a triazine herbicide that is applied for the pre- and post-emergence control of broadleaf and grassy weeds in cultivations of crops such as maize and sugarcane (Rodrigues and Almeida, 2011). The action of this compound blocks the electron flow in photosystem II (PSII), leading to the inhibition of CO_2_ assimilation and the generation of large amounts of reactive oxygen species (Hess, 2000). Thus, atrazine application results in an overall growth inhibition of sensitive plant species, followed by leaf chlorosis and necrosis, which may lead to plant death (Rodrigues and Almeida, 2011). Plant species that are tolerant to atrazine are able to detoxify the herbicide through a range of mechanisms, such as glutathione, glutathione *S*-transferases, cytochrome P450 monooxygenases, and ABC transporters (Pang et al., 2012). The induction of an efficient antioxidant response is also regarded as an important feature for atrazine tolerance (Alla and Hassan, 2006).

Despite its widespread use for weed control in many countries, atrazine presents high persistence in the environment, leading to the contamination of food, soils, and water resources (Graymore et al., 2001; Bortoluzzi et al., 2006). For this reason, atrazine has been banned in European Union (Bethsass and Colangelo, 2006). Many studies have indicated the deleterious effects of atrazine on animal and plant species in aquatic ecosystems (Dalton and Boutin, 2010; Brain et al., 2012; Santos and Martinez, 2012; Flores et al., 2013), as well as on soil microbiota (Chen et al., 2015) and human health (Sathiakumar et al., 2011). Even in non-target tolerant plant species, atrazine accumulation has been shown to cause toxic responses, inducing oxidative stress and negatively affecting crop growth and productivity (Alla and Hassan, 2006; Li et al., 2012). In this scenario, research into technologies that minimize the deleterious impacts of atrazine without hindering its biological activity toward target weeds is of great relevance.

Nanotechnology has emerged as a field with promising applications in agriculture, including the development of nanodevices for the delivery of genes, fertilizers, phytohormones, and plant protection products (Chen and Yada, 2011; Ghormade et al., 2011; Khot et al., 2012; Campos et al., 2014; de Oliveira et al., 2014). A variety of formulations based on nanoparticles have been produced as carrier systems for pesticides, enabling slow release of the active ingredient and extension of its duration of action (reviewed by Kah et al., 2013; Kah and Hofmann, 2014). Other advantages associated with the use of nanoparticles include greater protection against premature degradation and enhanced uptake of the active ingredient by target species, as compared to conventional formulations (Kah et al., 2013; Kah and Hofmann, 2014). These features allow reductions in both pesticides dosage and application frequency, while at the same time decreasing environmental contamination and the risk of harming non-target organisms (Kah et al., 2013; Kah and Hofmann, 2014).

With the aim of minimizing the contamination of natural resources by atrazine, our research group has developed carrier systems for this herbicide based on diverse types of nanoparticles, including polymeric nanocapsules (Grillo et al., 2012, 2014; Pereira et al., 2014) and nanospheres (Pereira et al., 2014; Grillo et al., 2015a), and solid lipid nanoparticles (de Oliveira et al., 2015). In particular, nanocapsules prepared with poly(epsilon-caprolactone) (PCL), a biodegradable aliphatic polyester, have emerged as a carrier system for atrazine with potential for application in agriculture (Grillo et al., 2012; Pereira et al., 2014). *In vitro* assays demonstrated high efficiency of encapsulation of atrazine in the PCL nanocapsules, as well as high colloidal stability of the nanoformulations and a modified release profile of the herbicide (Grillo et al., 2012; Pereira et al., 2014). Genotoxicity (using *Allium cepa* and human cells), cytogenetic (with human cells), and ecotoxicological tests (using the alga *Pseudokirchneriella subcapitata*) indicated a reduced toxicity of PCL nanocapsules containing atrazine toward non-target organisms, as compared to the free herbicide (Grillo et al., 2012; Clemente et al., 2014; Pereira et al., 2014). However, the biological activity of atrazine against target plants was maintained or even increased (Pereira et al., 2014; Oliveira et al., 2015). In a recent study, we analyzed the effects of atrazine-carrying PCL nanocapsules on the biochemical, physiological, and growth parameters of mustard plants (Oliveira et al., 2015). We demonstrated that encapsulation not only maintained the mechanism of action of atrazine, but also potentiated its post-emergence herbicidal activity against this target species, as compared to the effects of a commercially available atrazine product. As a result, a 10-fold reduction of the atrazine dosage was achieved, without compromising the biological activity of the herbicide. A greater pre-emergence herbicidal activity of atrazine-containing PCL nanocapsules against mustard seedlings has also been shown (Pereira et al., 2014). Therefore, we decided to investigate whether these nanoformulations might have any deleterious effects on non-target crops. This is an essential test before such systems can be recommended for safe use in agriculture.

In the present study, we evaluated the effects of pre- and post-emergence treatments with PCL nanocapsules containing atrazine on growth, physiological and oxidative stress parameters of maize (*Zea mays* L.) plants, as compared to those induced by a commercial atrazine formulation. The effects of PCL nanocapsules without the herbicides were also determined. Overall, we observed that PCL nanocapsules containing or not atrazine did not lead to persistent deleterious effects in maize plants, indicating that the technique could offer a safe tool for weed control without affecting crop growth.

## Material and methods

### Preparation of PCL nanocapsules

Atrazine-loaded poly(ε-caprolactone) nanocapsules were prepared by a nanoprecipitation method, according to the protocol described by Grillo et al. (2012). This technique, based on interfacial polymer deposition, consists of mixing an organic phase into an aqueous phase. The organic phase was composed of 100 mg of polymer (PCL), 30 mL of organic solvent (acetone), 200 mg of oil (triglycerides of capric and caprylic acids, in the form of Myritol® 318), 40 mg of sorbitan monostearate surfactant (Span 60), and 10 mg of atrazine. The aqueous phase was composed of 30 mL of a solution containing 60 mg of polysorbate 80 surfactant (Tween 80). The organic phase was slowly inserted into the aqueous phase, under magnetic stirring at room temperature, and maintained under agitation for 10 min. Finally, the nanoparticle suspension was evaporated to a volume of 10 mL using a rotary evaporator, resulting in an atrazine concentration of 1 mg mL^−1^, and stored in amber flasks at room temperature (25°C). Herbicide-free nanoparticles were prepared according to the same procedure, but omitting the atrazine. Atrazine, poly(ε-caprolactone), Tween 80, and Span 60 were purchased from Sigma-Aldrich. All other reagents (analytical grade) used to prepare the PCL nanocapsules were purchased from local suppliers.

### Plant material and growth conditions

*Zea mays* L. (Itapuã 700 hybrid) was used as the non-target crop model. The seeds, purchased from Isla Sementes (Porto Alegre, Brazil), were sown in plastic pots (10.5 cm high, 9.5 cm lower diameter, 14 cm upper diameter) filled with 1 kg of a mixture of clay soil and vermiculite (3:1). The soil was the same Rhodic Ferralsol as used in our previous study (Oliveira et al., 2015). The substrate was supplemented with 50 mL of complete Hoagland and Arnon's (1950) nutrient solution, on a weekly basis. Throughout the cultivation (14 days until treatments), the plants were kept in a greenhouse under natural conditions of light and temperature. The experiments were carried out from October to March (spring-summer). The average daily values of temperature, relative humidity, and accumulated global solar radiation were 23.7 ± 2.6°C, 74.4 ± 14.9%, and 18.9 ± 5.6 MJ m^−2^, respectively (data kindly provided by the Laboratory of Agrometeorology, Embrapa Soja, Londrina).

### Post-emergence assays

For post-emergence assays, four individuals were retained per pot after germination. Fourteen-days-old maize plants were treated with the following formulations: distilled water (control), nanocapsules without atrazine (NC), Gesaprim® 500 CG (Syngenta) containing atrazine at 1 mg mL^−1^ (ATZ), and nanocapsules containing atrazine at 1 mg mL^−1^ (NC+ATZ). Each pot was sprayed with 3.1 mL of the test sample, resulting in application of the atrazine dosage recommended by the manufacturer (2000 g atrazine per hectare). Treatments with commercial atrazine and nanoformulations diluted 10-fold in water were also performed (equivalent to 200 g atrazine per hectare), since NC+ATZ was previously shown to maintain the herbicidal activity against mustard plants at this lower dosage (Oliveira et al., 2015). The treatments were applied between 08:00 and 09:00 am. Macroscopic symptoms in the leaves were recorded using a Samsung ST200F camera. The physiological and oxidative stress parameters were determined 1, 2, 4, and 8 days after treatment. The dry weight analysis was measured at harvest.

### Pre-emergence assays

For pre-emergence assays, six seeds were sown per pot. The pots were then sprayed with the same formulations described for the post-emergence assays. The physiological, oxidative stress, and dry weight analyses were carried out 3 weeks after emergence of the plants.

### Physiological, oxidative stress, and dry weight characterization

Chlorophyll *a* fluorescence parameters were measured before dawn using an OS1p fluorometer (Opti-Sciences, Hudson, NH, USA). The maximum quantum yield of the PSII photochemistry was expressed as *F*_*v*_/*F*_*m*_ = (*F*_*m*_ – *F*_0_)/*F*_*m*_ (Oliveira et al., 2015). Leaf gas exchange parameters (net photosynthesis, stomatal conductance, intercellular CO_2_ concentration, and transpiration) were measured between 08:00 and 10:00 am using a Portable Photosynthesis System (LI-6400XT, LI-COR Biosciences, Lincoln, NE, USA). The infrared gas analyzer (IRGA) was connected to the 6400-02B measuring chamber, where the leaves were exposed to a saturating PAR (1500 μmol m^−2^ s^−1^). Lipid peroxidation was analyzed as a marker of oxidative stress. Freshly collected leaves (100 mg) were homogenized with cold TCA (0.2%) diluted in methanol, and then centrifuged at 10,000 × *g* for 5 min. The supernatant was used for determination of the MDA content by the thiobarbituric acid reactive substances (TBARS) method (Camejo et al., 1998). For weight analysis, shoots were harvested and kept for 72 h at 60°C, prior to dry weight measurement.

### Statistical analysis

Sixteen biological replicates were used for weight analysis, nine for gas exchange experiments, and five for chlorophyll fluorescence and oxidative stress analyses. For each time point, the data were analyzed using One-Way ANOVA followed by the Tukey post-test (*P* < 0.05).

## Results

### Post-emergence assays

**Figure 1:**
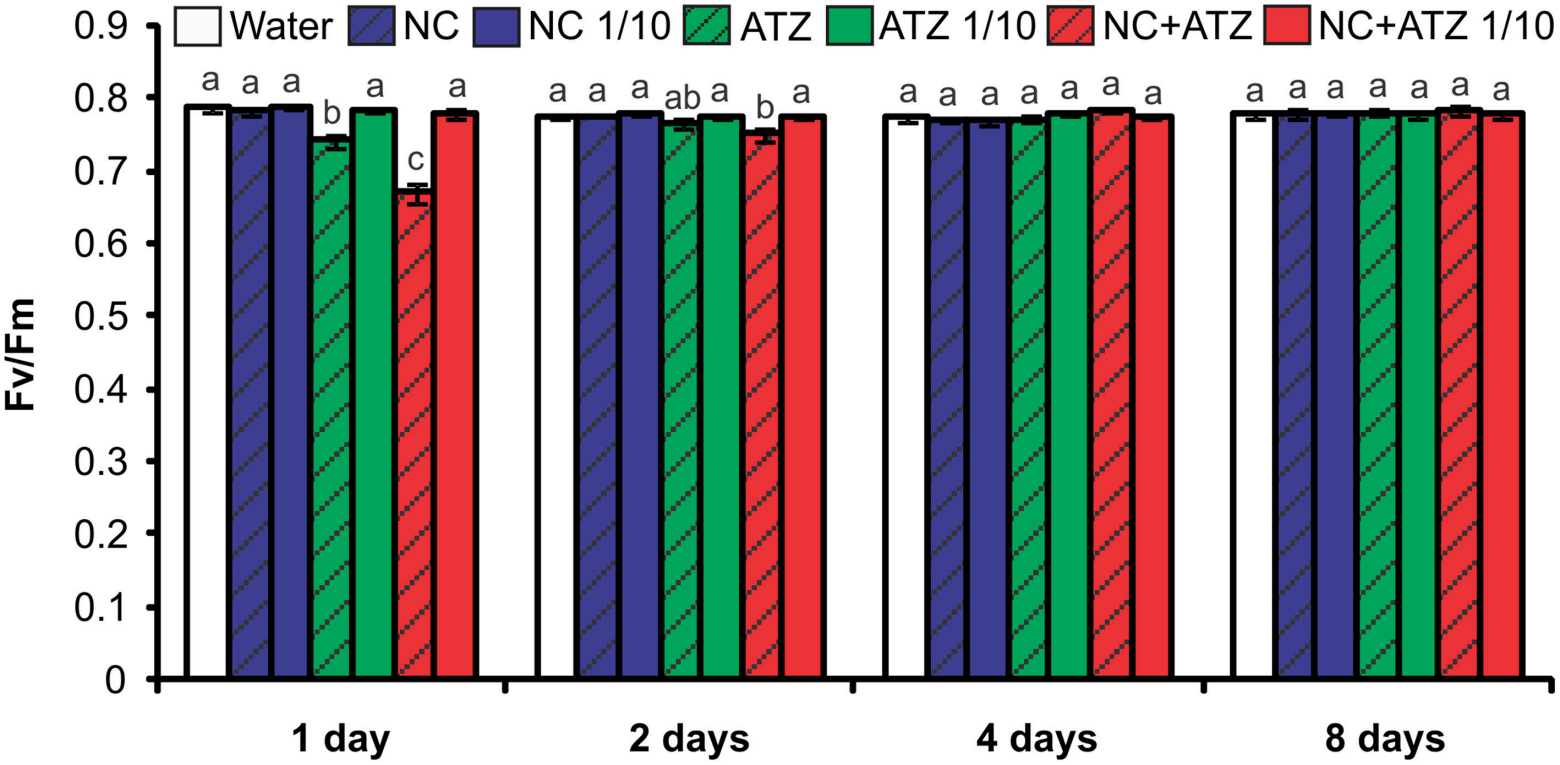
**Maximum photosystem II quantum yields of maize plants submitted to post-emergence treatment with the formulations**. Chlorophyll fluorescence parameters were evaluated 1, 2, 4, and 8 days after the plants were sprayed with 3.1 mL of water, empty PCL nanocapsules (NC), commercial atrazine (ATZ), or PCL nanocapsules containing atrazine (NC+ATZ). The formulations containing atrazine at 1 mg mL^−1^ were used undiluted or after 10-fold dilution in water (1/10), resulting in atrazine application dosages of 2000 or 200 g ha^−1^, respectively. Different letters for each time point indicate significantly different values, according to One-Way ANOVA followed by Tukey's test (*P* < 0.05). Data are shown as means ± SE (*n* = 5).

Both commercial atrazine (ATZ) and PCL nanocapsules containing atrazine (NC+ATZ) decreased the maximum quantum yield of PSII 1 day after the treatment of maize plants, as compared to control plants (Figure 1). These results are coherent with the inhibitory action of atrazine in PSII. Greater inhibition was caused by NC+ATZ (*F*_*v*_/*F*_*m*_ = 0.67), as compared to ATZ (*F*_*v*_/*F*_*m*_ = 0.74). However, the effects of the two formulations were transient, because the *F*_*v*_/*F*_*m*_ ratios for plants treated with ATZ or NC+ATZ recovered to the same values obtained for control plants 2 or 4 days after treatment, respectively. When 10-fold diluted atrazine-containing formulations (ATZ 1/10 and NC+ATZ 1/10) were applied, no effects on PSII photochemistry were observed, even after shorter times. Regardless of the dilution, the treatments with nanocapsules without atrazine (NC and NC 1/10) also had no effect on the *F*_*v*_/*F*_*m*_ ratio of the maize plants.

**Figure 2:**
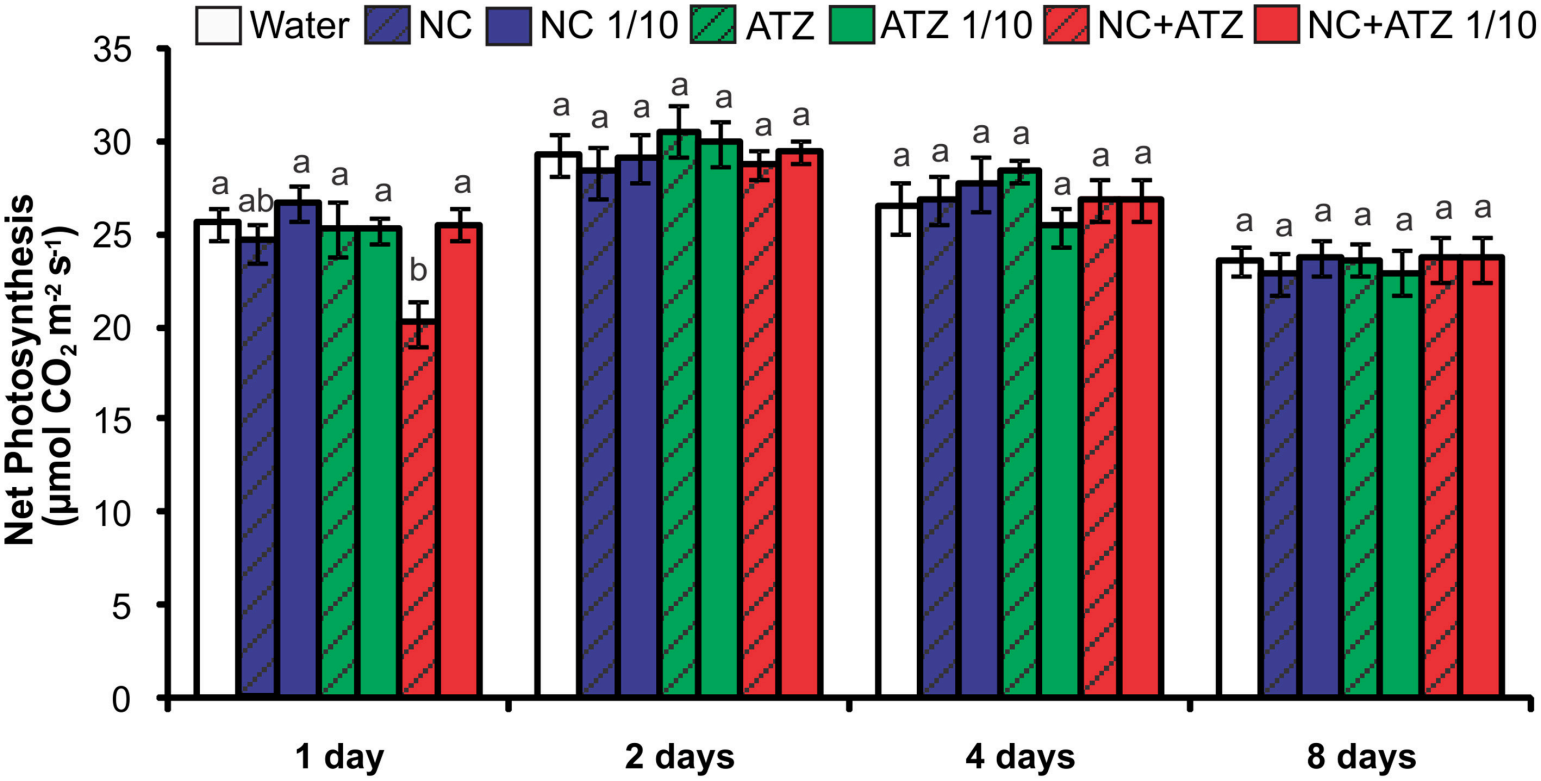
**Net photosynthesis of maize plants submitted to post-emergence treatment with the formulations**. Net photosynthesis was evaluated 1, 2, 4, and 8 days after the plants were sprayed with 3.1 mL of water, empty PCL nanocapsules (NC), commercial atrazine (ATZ), or PCL nanocapsules containing atrazine (NC+ATZ). The formulations containing atrazine at 1 mg mL^−1^ were used undiluted or after 10-fold dilution in water (1/10), resulting in atrazine application dosages of 2000 or 200 g ha^−1^, respectively. Different letters for each time point indicate significantly different values, according to One-Way ANOVA followed by Tukey's test (*P* < 0.05). Data are shown as means ± SE (*n* = 9).

The only treatment that negatively affected the net photosynthetic CO_2_ assimilation rate of the plants was NC+ATZ (Figure 2). This effect was only observed on the day after the plants were sprayed with NC+ATZ, since from 2 days after NC+ATZ treatment onwards no differences in net photosynthesis were detected, as compared to control plants. Stomatal conductance, intercellular CO_2_ concentration, and transpiration were not affected by any of the formulations tested (data not shown).

**Figure 3:**
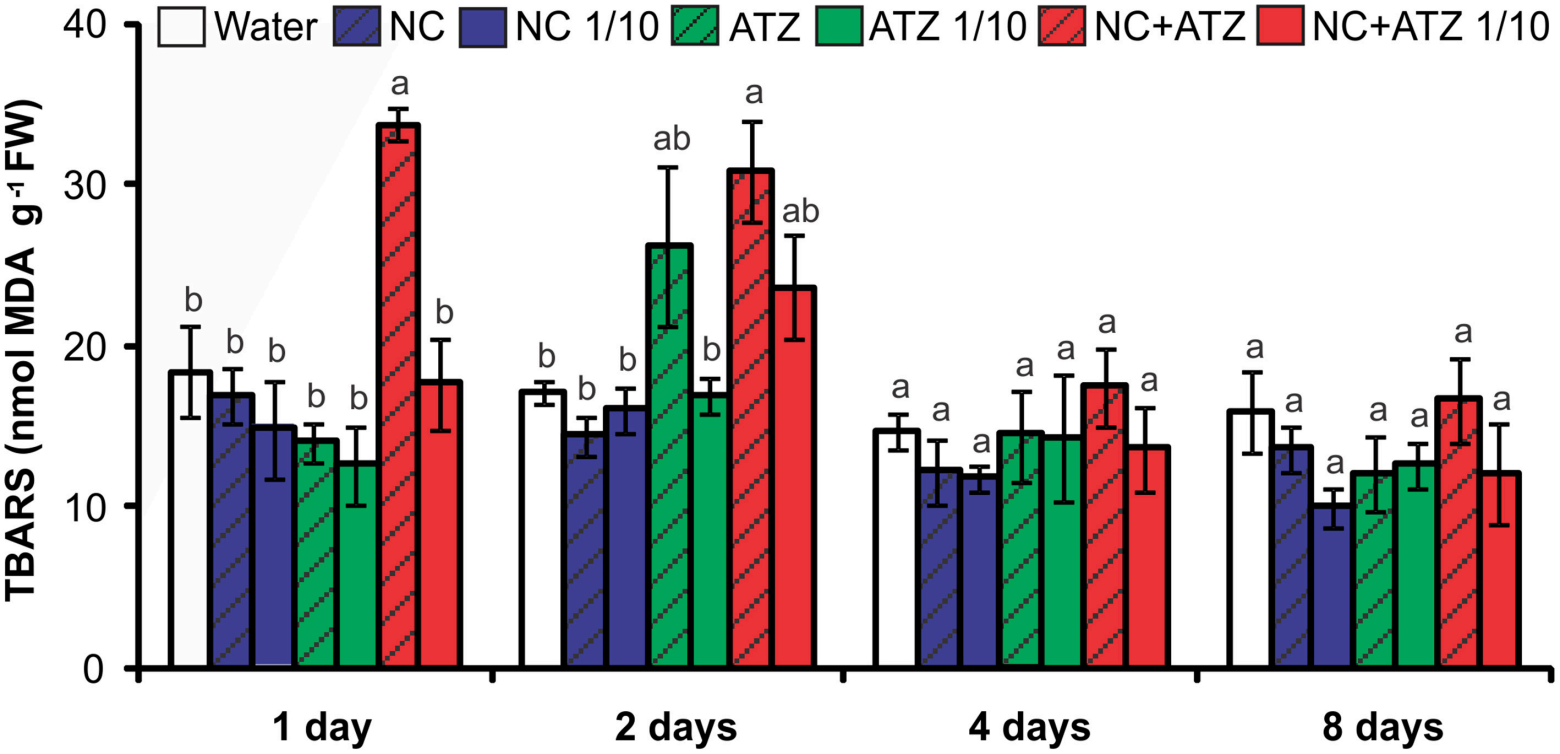
**Leaf lipid peroxidation of maize plants submitted to post-emergence treatment with the formulations**. Lipid peroxidation was evaluated 1, 2, 4, and 8 days after the plants were sprayed with 3.1 mL of water, empty PCL nanocapsules (NC), commercial atrazine (ATZ), or PCL nanocapsules containing atrazine (NC+ATZ). The formulations containing atrazine at 1 mg/mL were used undiluted or after 10-fold dilution in water (1/10), resulting in atrazine application dosages of 2000 or 200 g ha^−1^, respectively. Different letters for each time point indicate significantly different values, according to One-Way ANOVA followed by Tukey's test (*P* < 0.05). Data are shown as means ± SE (*n* = 5).

The application of NC+ATZ initially enhanced lipid peroxidation in maize leaves, as compared to the controls (Figure 3). After 1 and 2 days following treatment with NC+ATZ, leaf MDA content was 33.8 ± 1.1 and 30.9 ± 3.1 nmol g^−1^, respectively, while in water-sprayed leaves it was 18.5 ± 2.9 and 17.3 ± 0.7 nmol g^−1^. From 4 days after treatment onwards, the MDA content of leaves sprayed with NC+ATZ returned to control levels. No significant effects on leaf lipid peroxidation were induced by the other formulations tested (NC, NC 1/10, ATZ, ATZ 1/10, and NC+ATZ 1/10).

**Figure 4:**
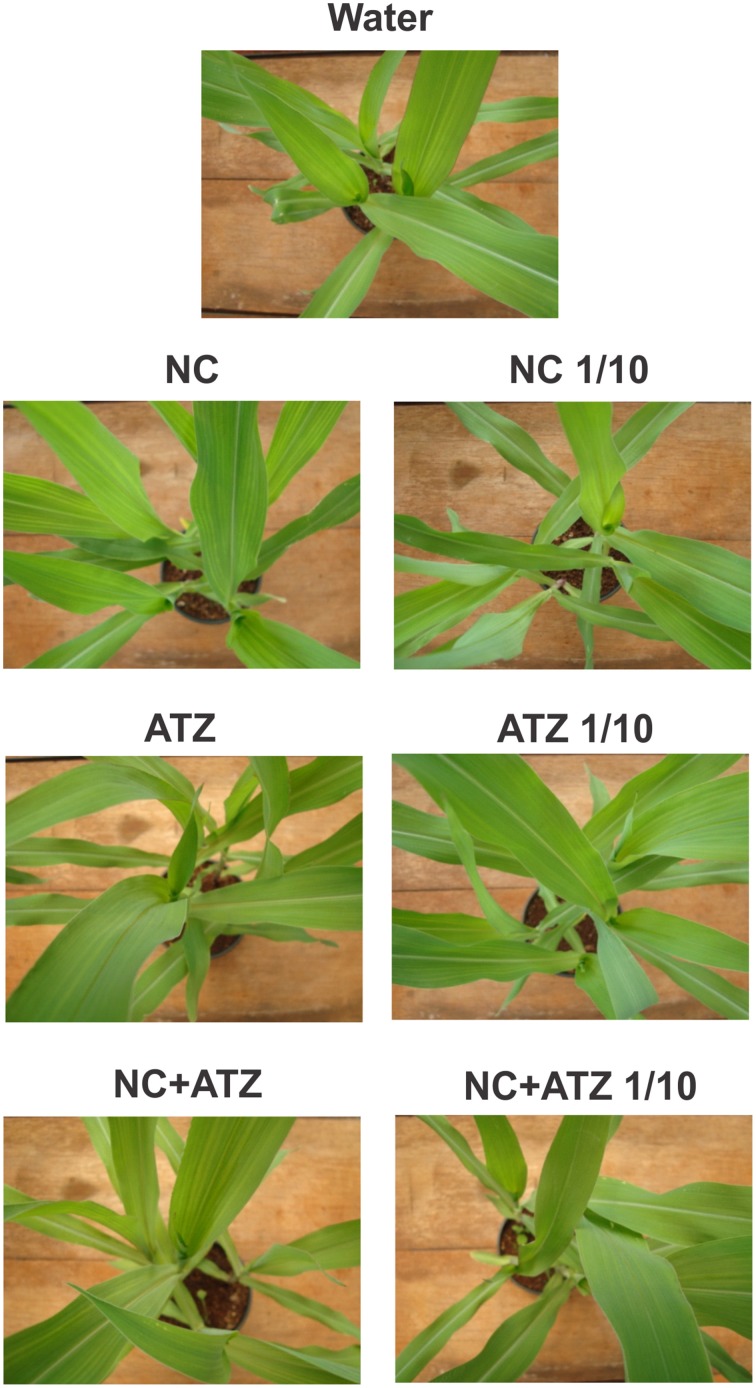
**Symptom evolution in maize leaves submitted to post-emergence treatment with the formulations**. Symptoms were recorded 8 days after the plants were sprayed with 3.1 mL of water, empty PCL nanocapsules (NC), commercial atrazine (ATZ), or PCL nanocapsules containing atrazine (NC+ATZ). The formulations containing atrazine at 1 mg mL^−1^ were used undiluted or after 10-fold dilution in water (1/10), resulting in atrazine application dosages of 2000 or 200 g ha^−1^, respectively.

**Figure 5:**
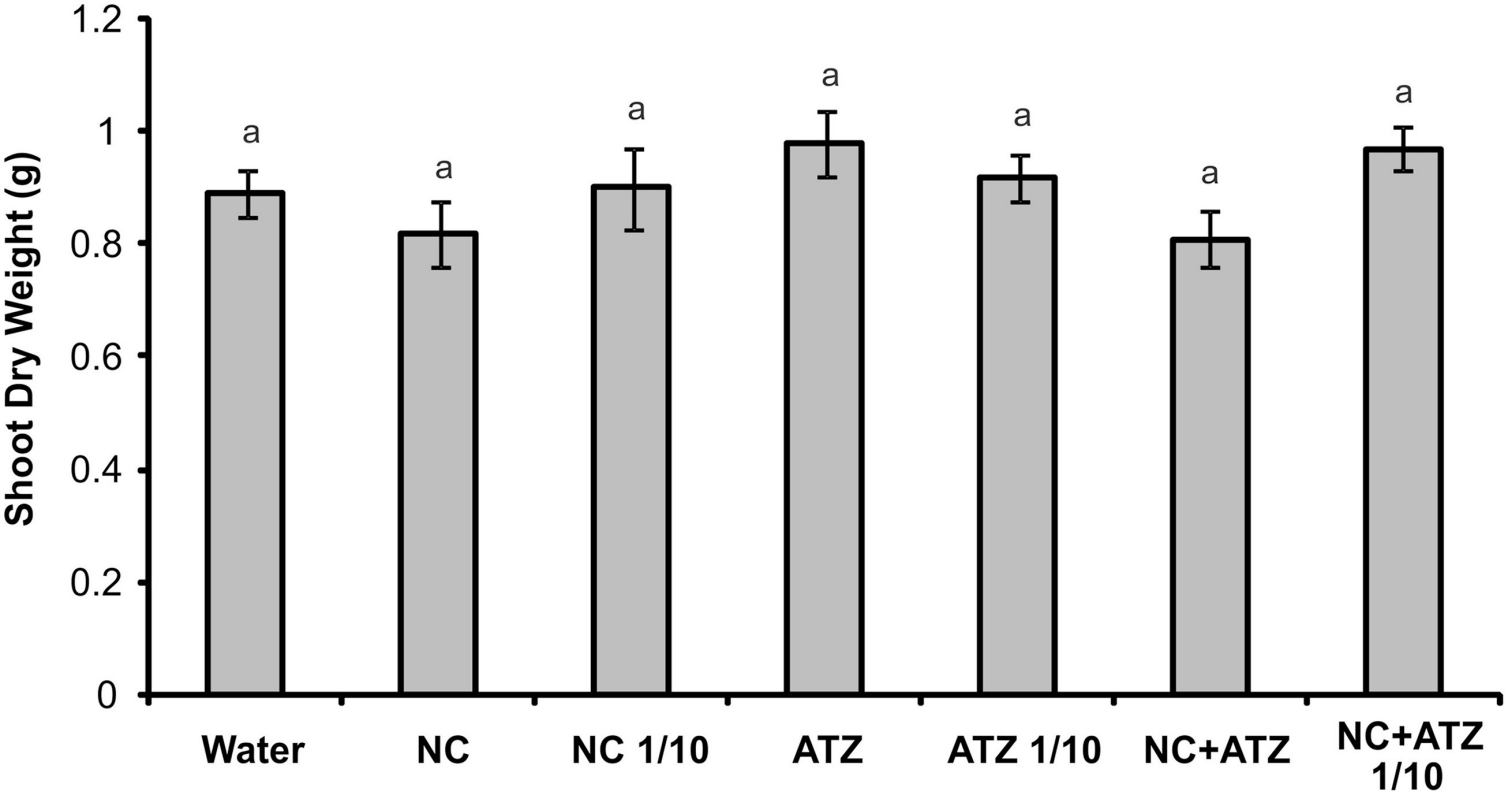
**Shoot dry weight of maize plants submitted to post-emergence treatment with the formulations**. Shoots were sampled 8 days after the plants were sprayed with 3.1 mL of water, empty PCL nanocapsules (NC), commercial atrazine (ATZ), or PCL nanocapsules containing atrazine (NC+ATZ). The formulations containing atrazine at 1 mg mL^−1^ were used undiluted or after 10-fold dilution in water (1/10), resulting in atrazine application dosages of 2000 or 200 g ha^−1^, respectively. Equal “a” letters indicated that no significant differences among the treatments were detected in One-Way ANOVA test (*P* = 0.21). Data are shown as means ± SE (*n* = 16).

No macroscopic symptoms were observed in leaves sprayed with any of the formulations (Figure 4). Accordingly, none of the tested formulations affected the shoot dry weight of the maize plants (Figure 5).

### Pre-emergence assays

**Figure 6:**
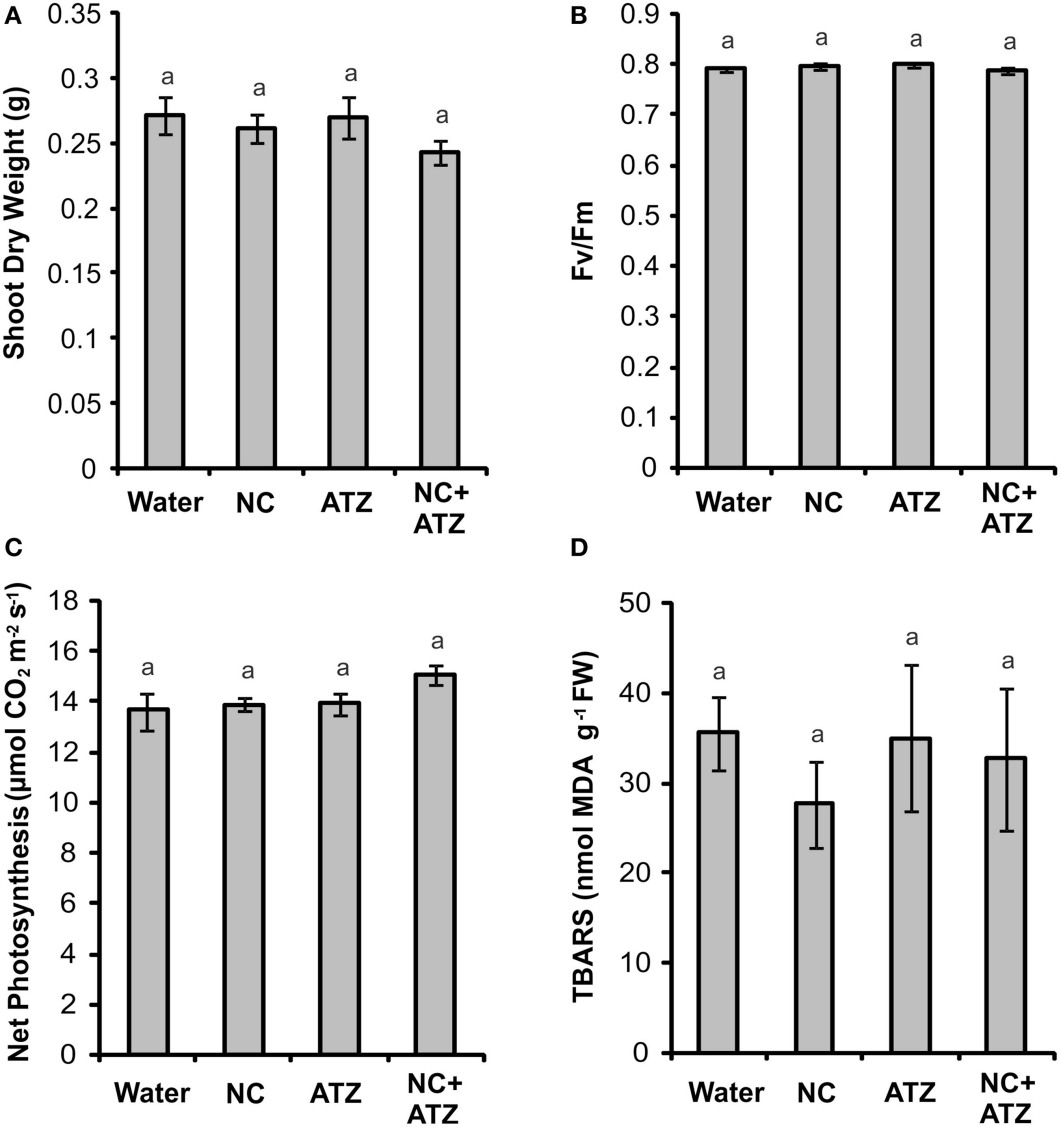
**Effect on maize plants of pre-emergence treatment with the formulations**. Maize seeds were germinated in a soil:vermiculite mixture sprayed with 3.1 mL of water, empty PCL nanocapsules (NC), commercial atrazine (ATZ), or PCL nanocapsules containing atrazine (NC+ATZ). The formulations contained atrazine at 1 mg mL^−1^, resulting in a dosage of 2000 g ha^−1^. Shoot dry weight **(A)**, maximum quantum yield of photosystem II **(B)**, net photosynthesis **(C)**, and leaf lipid peroxidation **(D)** were evaluated 3 weeks after emergence of the plants. Equal “a” letters indicated that no significant differences among the treatments were detected in One-Way ANOVA test (**A**: *P* = 0.42; **B**: *P* = 0.11; **C**: *P* = 0.19; **D**: *P* = 0.73). Data are shown as means ± SE (**A**: *n* = 16; **B**: *n* = 5; **C**: *n* = 9; **D**: *n* = 5).

Pre-emergence treatment with NC, ATZ, or NC+ATZ did not lead to any effects on shoot dry weight (Figure 6A), maximum quantum yield of PSII (Figure 6B), net photosynthesis (Figure 6C), or leaf lipid peroxidation (Figure 6D) of the maize plants, as compared to the controls.

## Discussion

PCL nanocapsules have emerged as an efficient carrier system for atrazine (Grillo et al., 2012, 2014; Pereira et al., 2014). In addition to reducing the cytogenotoxic effects of atrazine (Grillo et al., 2012; Clemente et al., 2014; Pereira et al., 2014), we have shown that the nanoencapsulation of this herbicide effectively increases its pre- and post-emergence herbicidal activity against mustard plants, a target species (Pereira et al., 2014; Oliveira et al., 2015). In the present study, we evaluated whether the pre- or post-emergence application of PCL nanocapsules containing atrazine would negatively affect maize plants, a non-target crop in whose cultivations atrazine is widely applied. Although some acute and transient effects on photosynthetic and oxidative stress parameters were detected in maize leaves after post-emergence treatment with atrazine-loaded PCL nanocapsules, these effects were not persistent and did not affect shoot growth. In the case of pre-emergence treatment, none of the analyzed parameters was affected by the nanoformulation. The results therefore provide further insight into the use of PCL nanocapsules containing atrazine as a safe tool for weed control without affecting crop growth.

Previous studies have shown that atrazine induces toxic responses even in tolerant non-target crops (Alla and Hassan, 2006; Li et al., 2012; Zhang et al., 2012, 2014). In the case of maize plants, post-emergence application of atrazine may induce oxidative stress, alter gene expression, and even inhibit plant growth, depending on the cultivar and the dosage applied (Alla and Hassan, 2006; Li et al., 2012; Pang et al., 2012). Here, in post-emergence tests, PCL nanocapsules containing atrazine, when applied at the standard field dosage (2000 g ha^−1^), initially led to an increase in leaf lipid peroxidation (Figure 3) and decreases in net CO_2_ assimilation (Figure 2) and maximum quantum yield of PSII (Figure 1). However, all of these parameters were unaffected 4 and 8 days after the application of encapsulated atrazine (Figures 1–3). These results suggest that the negative effects of atrazine were transient, probably due to the ability of maize plants to detoxify the herbicide (Pang et al., 2012) and to induce an effective antioxidant response (Alla and Hassan, 2006; Li et al., 2012).

When a commercial atrazine formulation was applied at the same dosage, an inhibitory effect was only observed on PSII efficiency (Figure 1), indicating the induction of a weaker response than that elicited by encapsulated atrazine. This finding is consistent with the results of previous phytotoxicity assays, which showed that atrazine-containing PCL nanocapsules exerted a greater effect on mustard plants, as compared to commercially available atrazine (Oliveira et al., 2015). However, it is noteworthy that treatment with atrazine-loaded PCL nanocapsules or with commercial atrazine at the recommended dosage did not result in the development of any macroscopic symptoms in the maize leaves (Figure 4), or in any effects on shoot growth (Figure 5). Hence, the initial and transient response induced by the post-emergence application of the herbicide did not compromise growth of the maize cultivar analyzed here. In the case of the pre-emergence assays, neither the physiological nor the growth parameters were affected by atrazine, whether free or encapsulated (Figure 6).

Due to its higher efficacy than the commercial formulation, the application of an atrazine-containing PCL nanocapsules formulation at a 10-fold lower concentration does not compromise its post-emergence herbicidal activity against target plants (Oliveira et al., 2015). Here, when 10-fold diluted nanoencapsulated atrazine was applied to the maize plants, no effects were detected even after shorter periods of time, the same observed for plants treated with 10-fold diluted commercial atrazine (Figures 1–5). Taken together, these results suggest that post-emergence treatment with atrazine-containing PCL nanocapsules at 200 g ha^−1^ (10-fold lower than the recommended dosage) would still be effective for weed control, without eliciting any toxic response in a non-target crop. Additionally, it would imply that reduced inputs of atrazine could be used in agricultural systems, potentially decreasing contamination of the environment with the herbicide (Oliveira et al., 2015).

Another important aspect that has emerged with the development of nanopesticides is the need to evaluate the effects of nanomaterials *per se* on plants (Rico et al., 2011). Studies have shown that carbon nanoparticles can exert effects on various plant species (Chen et al., 2010; Cifuentes et al., 2010; Djikanović et al., 2012). As an example, Khodakovskaya et al. (2009) showed that carbon nanotubes affect seed germination and the development of tobacco and tomato plants. In the present study, none of the analyzed parameters of the maize plants were affected by the pre- or post-emergence treatment with nanocapsules without atrazine (Figures 1–6), excluding the possibility of phytotoxic effects of PCL nanocapsules *per se*. These findings are in agreement with the results of post-emergence assays using mustard plants (Oliveira et al., 2015). Differently, in pre-emergence treatments, PCL nanocapsules without atrazine were shown to decrease the germination index of mustard plants, an effect that might have arisen from interaction of the nanocapsules with the seed tegument (Pereira et al., 2014).

Given that PCL is a biodegradable polymer, it should not be harmful to the environment (Woodruff and Hutmacher, 2010). Nevertheless, the environmental fate of polymeric nanocapsules and other nanomaterials is still under debate, and the environmental risks associated with the application of nanopesticides need further clarification (Kah et al., 2013; Kookana et al., 2014). Factors that can influence nanopesticide bioavailability and toxicity include particle size distribution, particle number concentration, surface charge, release rate, and the ratio between the free and nanoparticle-loaded pesticide fractions (Kookana et al., 2014). In addition, environmental variables such as pH, ionic strength, light, temperature, microorganisms, and natural organic matter can also change the degree of dispersion or agglomeration of nanomaterials over time, modifying their fate and behavior in the environment (Mohd et al., 2014; Wagner et al., 2014; Grillo et al., 2015a,b). The literature reports several studies concerning the bioavailability, toxicity, and fate of atrazine in the environment [Environmental Protection Agency (EPA), 2012]; however, investigations involving nanopesticides remain scarce (Kah and Hofmann, 2014). In a previous study, Clemente et al. (2014) showed that atrazine-loaded PCL nanocapsules presented lower toxicity to the green alga *P. subcapitata*, as compared to free atrazine, suggesting that use of the nanoformulation might have environmental benefits.

Knowledge of the processes of sorption and degradation of nanopesticides is important in order to be able to predict the fate of pesticides in soils (Kah et al., 2014). Pereira et al. (2014) showed that the encapsulation of atrazine in PCL nanoparticles reduced the mobility of the herbicide in soil. In another study, Kah et al. (2014) discussed the issues of regulatory protocols and the fate and properties of atrazine-loaded PCL nanocapsules in soils, and it was noted that the use of a nanopesticide formulation affected the fate of atrazine. Further investigations of the bioavailability and persistence of nanopesticides are therefore required in order to understand the interactions involved.

This study provides information concerning the interactions between nanopesticides and plants. The findings indicated that the use of atrazine-loaded PCL nanocapsules did not lead to persistent side effects in a non-target crop species, and could therefore provide a safe tool for weed control without affecting crop growth. This conclusion may be further substantiated by future studies focusing on the effects of the nanoformulations on maize plants growing under different stress conditions, as well as analyzing additional parameters of plants (e.g., pigment content, oxygen evolution, cell death). Moreover, a comprehensive study designed to investigate the underlying mechanism of action of nanopesticides in plants is currently in preparation.

## Funding

Fundação de Amparo à Pesquisa do Estado de São Paulo (LFF: #2013/12322-2; RG: #2011/01872-6), Conselho Nacional de Desenvolvimento Científico e Tecnológico, Coordenação de Aperfeiçoamento de Pessoal de Nível Superior, and INCT-TA/CNPq (CBRM: 573949/2008-5).

### Conflict of interest statement

The authors declare that the research was conducted in the absence of any commercial or financial relationships that could be construed as a potential conflict of interest.
